# TREM-1 as a potential prognostic biomarker associated with immune infiltration in clear cell renal cell carcinoma

**DOI:** 10.1186/s12957-023-03013-w

**Published:** 2023-05-23

**Authors:** Yaling Pu, Danyang Cai, Lingling Jin, Fenfen Xu, Enru Ye, Lina Wu, Licai Mo, Suzhi Liu, Qunyi Guo, Gang Wu

**Affiliations:** 1 Taizhou Hospital of Zhejiang Province, Shao Xing University, No. 150 Ximen Street, TaizhouZhejiang, 317000 Linhai China; 2 Department of Radiation Oncology, Taizhou Hospital of Zhejiang Province Affiliated to Wenzhou Medical University, No. 150 Ximen Street, TaizhouZhejiang, 317000 Linhai China; 3 Department of Pathology, Taizhou Hospital of Zhejiang Province Affiliated to Wenzhou Medical University, No. 150 Ximen Street, TaizhouZhejiang, 317000 Linhai China; 4 Department of Pharmacy, Taizhou Hospital of Zhejiang Province Affiliated to Wenzhou Medical University, No. 150 Ximen Street, TaizhouZhejiang, 317000 Linhai China; 5 Department of Pathology, Enze Hospital, Taizhou Enze Medical Center, No. 150 Ximen Street, TaizhouZhejiang, 317000 Linhai China; 6 Department of Urology, Taizhou Hospital of Zhejiang Province Affiliated to Wenzhou Medical University, No. 150 Ximen Street, TaizhouZhejiang, 317000 Linhai China; 7 Department of Neurology, Taizhou Hospital of Zhejiang Province Affiliated to Wenzhou Medical University, No. 150 Ximen Street, TaizhouZhejiang, 317000 Linhai China; 8 Department of Hematology and Oncology, Taizhou Hospital of Zhejiang Province Affiliated to Wenzhou Medical University, No. 150 Ximen Street, TaizhouZhejiang, 317000 Linhai China

**Keywords:** Immune-related signature, Clear cell renal cell carcinoma, The Cancer Genome Atlas, Tumor immune microenvironment, TREM-1

## Abstract

**Background:**

The tumor immune microenvironment plays a crucial role in the efficacy of various therapeutics. However, their correlation is not yet completely understood in Clear cell renal cell carcinoma (ccRCC). This study aimed to investigate the potential of TREM-1 as a potential novel biomarker for ccRCC.

**Methods:**

We constructed a ccRCC immune prognostic signature. The clinical characteristics, the status of the tumor microenvironment, and immune infiltration were analyzed through the ESTIMATE and CIBERSORT algorithms for the hub gene, while the Gene Set Enrichment Analysis and PPI analysis were performed to predict the function of the hub gene. Immunohistochemical staining was used to detect the expression of TREM-1 in renal clear cell carcinoma tissues.

**Results:**

The CIBERSORT and ESTIMATE algorithms revealed that TREM-1 was correlated with the infiltration of 12 types of immune cells. Therefore, it was determined that TREM-1 was involved in numerous classical pathways in the immune response via GSEA analysis. In Immunohistochemical staining, we found that the expression of TREM-1 was significantly upregulated with increasing tumor grade in renal clear cell carcinoma, and elevated TREM-1 expression was associated with poor prognosis.

**Conclusions:**

The results suggest that TREM-1 may act as an implicit novel prognostic biomarker in ccRCC that could be utilized to facilitate immunotherapeutic strategy.

## Introduction

Globally, clear cell renal cell carcinoma (ccRCC) is one of the most common pathological types of renal cell carcinoma (RCC), accounting for about 75% of RCCs [1]. Although ccRCC can be detected in its early stages and surgical resection is the mainstream strategy for its mitigation, up to one-third of patients with ccRCC will still develop metastases [2]. ccRCC is a highly immune-invasive tumor. Immunotherapy-based combination therapies have now become the standard of care, so the use of immune checkpoint inhibitors (ICIs) to mitigate tumor-associated suppressed anti-cancer immune responses, such as programmed cell death-1 (PD-1) and cytotoxic T lymphocyte-associated antigen-4 (CTLA-4), has revolutionized the treatment of advanced ccRCC and significantly improved survival status in patients with this disease [3, 4]. Despite advances in treatment, patients with advanced ccRCC still have a poor prognosis, mainly due to drug resistance [5]. This response often relies on the dynamic interaction between the tumor and the tumor microenvironment (TME) [6]. However, the molecular mechanisms and pathological processes involved in cancer-causing immunity of ccRCC are unknown.

The TME, which includes tumor cells, local stromal cells, and other immune cells [7], is crucial in tumor progression and metastasis. Moreover, most non-tumor cell populations, such as stromal and immune cells, have been identified as prognostic markers of tumorigenesis. Previous studies have demonstrated that inflammation in TME, in which infiltrated diverse inflammatory cells contribute to tumorigenesis, is generally considered as a hallmark of cancer [8, 9]. Cancer immunotherapy mainly exerts effects with some proteins to enhance the function of or restore immune cells in the TME. Considering the importance of immunity in the development and the treatment of cancer, discovering novel ccRCC immune-related biomarkers may be useful for immunotherapy.

TREM-1 is involved in triggering DAP12 association and Src family kinase-mediated phosphorylation of ITAM tyrosine on cell membranes [10–12], followed by recruitment and phosphorylation of GRBP-2, Syk, and ZAP70, thereby initiating downstream signal transduction [10, 11, 13]. These pathways induce the mobilization of Ca2+, actin cytoskeletal rearrangement, and activation of transcription factors such as Elk1 (ETS domain protein), NFAT (nuclear factor that activates T cells), AP1, c-Fos, c-Jun, and Nf-κB, which transcribe genes encoding pro-inflammatory cytokines, chemokines, and cell surface molecules [14]. Recent studies have shown a close link between TREM-1 and cancer, as TREM-1 is selectively expressed on tumor-associated macrophages (TAMs) in non-small cell lung cancer [15], activates in the liver Kupffer cells, and promotes the progression of hepatocellular carcinoma [16]. Moreover, the increased abundance of TREM-1 in neutrophils may promote the development of colorectal cancer [17]. Nevertheless, the association of TREM-1 with immune infiltration in ccRCC has not been investigated.

In this paper, we identified the prognostic immune-related genes (IRGs) and immune-related lncRNAs (irlncRNAs) based on the TCGA database. Subsequently, differential expression analysis combined with Gene Ontology (GO), Kyoto Encyclopedia of Genes and Genomes (KEGG), and protein and protein interaction (PPI) analysis were performed to find the immune-related hub gene in high- and low-risk groups by utilizing the LASSO regression model. We have selected and compared IL-6, CXCL1, CXCL13, CXCL8, CXCL2, CXCL6, PI3, NFKBIZ, and TREM1 hub genes. Although these hub genes can secrete the release of promote inflammatory factors, affect the tumor microenvironment [18–22], only TREM-1 is a cell surface receptor and a member of the immunoglobulin superfamily that potently amplifies inflammatory responses by secretion of proinflammatory mediators. TREM-1 is known as an activating receptor expressed on neutrophils, monocytes, and macrophages [23]. Furthermore, we performed immune microenvironment analysis, immune cell infiltration analysis, clinical correlation analysis, and GSEA analysis for TREM-1 to determine their potential involvement in immune infiltration in ccRCC.

## Methods

### Data collection and pre-processing

The data of 539 ccRCC and 72 normal samples were obtained from the TCGA database (https://portal.gdc.cancer.gov/). From the ImmPort database (https://www.immport.org/home), a list of recognized 2484 IRGs was obtained. We have covered case-control studies of bioinformatics analysis methods.

### Establishment of weighted co-expression network and immune prognostic signature

The weighted gene co-expression network analysis (WGCNA) was used to identify the modules associated with survival time and status. We obtained a list of irlncRNAs that were co-expressed with the IRGs through Pearson correlation analysis [18]. Then, we conducted a univariate Cox analysis of the irlncRNAs to identify the prognostic irlncRNAs.

### *Construction of *the* prognostic IRGs and irlncRNA signature model*

To construct the prognostic signature model, the Lasso regression analysis was applied using the R package glmnet. The stability of the model was verified via univariate and multivariate Cox regression analysis in combination with the clinicopathological characteristics correlation analysis. Kaplan–Meier (KM) survival curves and receiver operating characteristic curves (ROC) were drawn using the survminer and timeROC R packages, respectively.

### *Differential gene *analysis* and functional enrichment analysis*

Differentially expressed IRGs were analyzed through the limma R package using the following screening criteria: false discovery rate (FDR) < 0.05 and log2 |fold change| >1, which means that the difference multiplier is greater than 2 times. Functional enrichment analyses using GO and KEGG were conducted for differentially expressed IRGs using the clusterProfiler R package.

### Exploring the hub gene and validation of its mRNA expression level and prognostic value

The hub gene was screened from the IRGs in the GO enrichment results using the String website (https://string-db.org/). The setting parameters of the String website: network type: full STRING network; meaning of network edges: evidence; active interaction sources: textmining, experiments, databases, co-expression, neighborhood, gene fusion, co-occurrence; minimum required interaction score: medium confidence (0.400); network display mode: interactive svg. Then, the mRNA expression levels of the hub gene in ccRCC were assessed in TIMER and TCGA database. The Kaplan–Meier (KM) survival curve, univariate, and multivariate Cox regression analyses were plotted to investigate the prognostic value using the corresponding R packages.

### Exploring the relationship between hub gene expression and immunocytes and their marker genes

The ESTIMATE algorithm was applied to evaluate the tumor microenvironment scores. Then, the corresponding survival analysis was performed using the survminer R package. To evaluate the relationship between the proportion of 22 immune cell types and the hub gene, CIBERSORT, a newly developed deconvolution algorithm, was applied. Then, the correlation between TREM-1 expression and the gene markers of the main immune cells was verified in TCGA.

### Gene set enrichment analysis and PPI network analysis of the hub gene

Gene set enrichment analysis (GSEA) was used to analyze the immune-related KEGG pathways of the hub gene through the R package. To further investigate the role of the hub gene, we constructed the PPI networks using the String website (https://www.string-db.org/) and GeneMANIA (http://genemania.org/).

### *Chemicals and *antibody

All chemical reagents were purchased from Beijing Sinopharm Chemical Reagents Co. Ltd. (Beijing, China). TREM-1 antibody was from Santa Cruz Biotechnology, Inc. (Cat#: sc-9013. Texas, USA).

### *Kidney cancer *tissue

The formalin-fixed, paraffin-embedded kidney cancer TMA used in this work was obtained from the Department of Pathology, Taizhou Hospital, Zhejiang Province from January 2021 to November. To avoid subjective differences, all cancer tissue samples were pathologically confirmed by two pathologists with 8 years of diagnostic experience in accordance with Fuhrman nuclear grading [24]. The use of human kidney cancer tissue samples was approved by the Institutional Ethic Committee of Taizhou Hospital of Zhejiang Province (K20210755).

Entry criteria: (1) patients with no metastasis found before surgery, (2) no history of major diseases or malignant tumors in the past, (3) the specimen collection method is total nephrectomy or partial nephrectomy, (4) postoperative or puncture biopsy confirmed clinicopathological confirmation as ccRCC.

Exclusion criteria: (1) patients with evidence of metastasis, who have received radiotherapy, chemotherapy, immunotherapy and molecularly targeted therapy; (2) patients with postoperative pathological confirmation of mixing two or more clinical pathological types; (3) patients with other major diseases after surgery.

### Immunohistochemical

Briefly, paraffin-embedded 3-μm-thick sections were deparaffinized, rehydrated, submerged into citric acid buffer for heat-induced antigen retrieval, immersed in 0.3% hydrogen peroxide to block endogenous peroxidase activity, blocked with 3% bovine serum albumin, incubated with primary antibodies at 4°C overnight, and developed using the DAKO ChemMate Envision Kit/HRP (Dako-Cytomation, USA). The sections were then counterstained with hematoxylin, dehydrated, cleared and mounted. The tissues exhibiting brown staining in the cytoplasm, nucleus, or membrane were considered positive. The slides were scanned using Leica SCN400 slide scanner (Leica Biosystems, Wetzlar, Germany). The optical density (OD) of protein expression was quantitatively determined using Image J software.

## Results

### Identification of prognostic IRGs and irlncRNAs

**Fig. 1:**
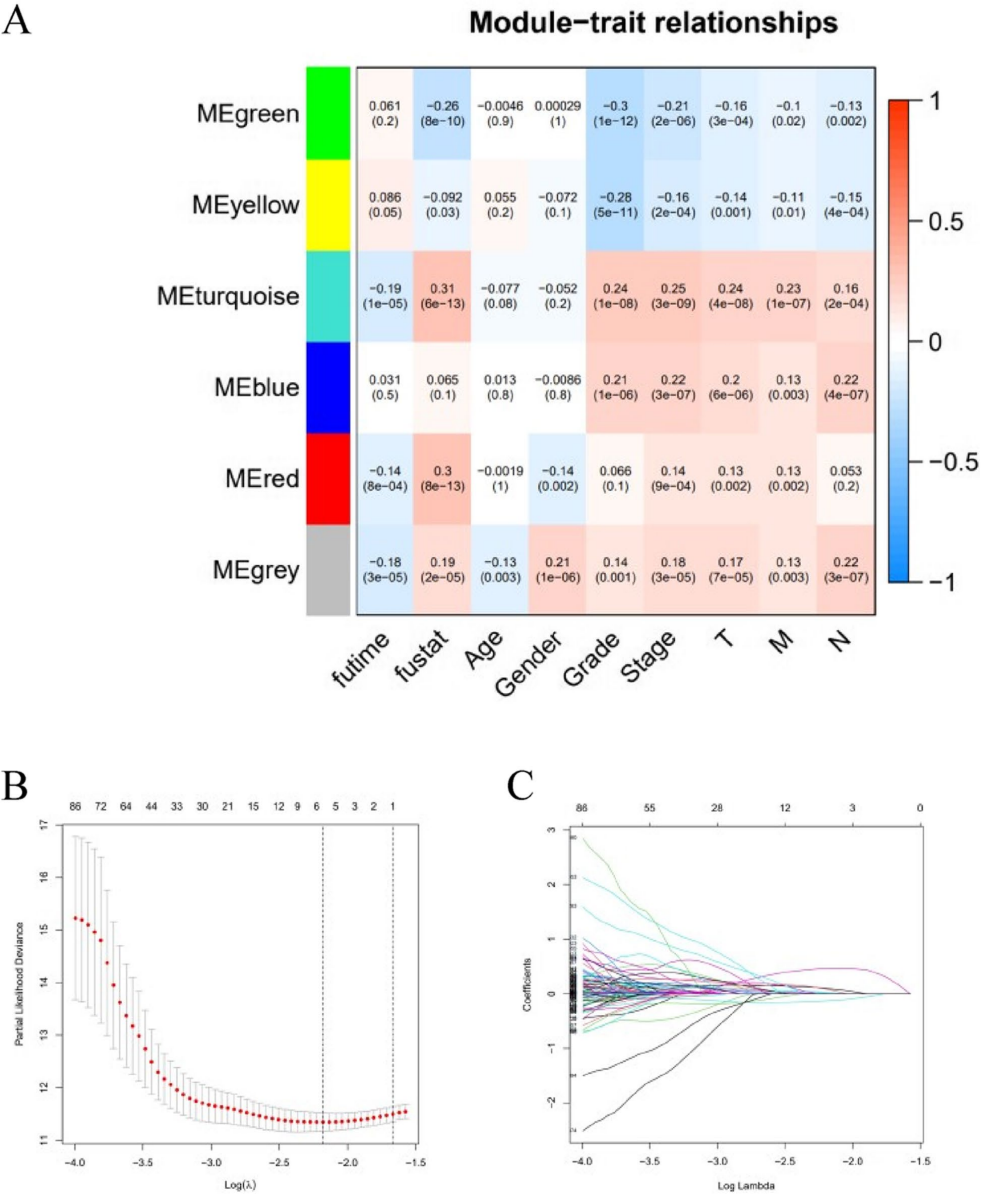
The WGCNA analysis and the LASSO Cox regression model. **A** The correlation coefficient and the *p* value are shown in the figure.** B–C** The parameters of LASSO Cox regression model

We identified 1358 differentially expressed IRGs associated with ccRCC using the limma package in the R language. Subsequently, the clinical data of ccRCC and 1358 IRGs were analyzed and classified through WGCNA analysis using the R language (Fig. 1A). A total of 200 prognostic IRGs were obtained by univariate Cox analysis (*p* < 0.05). Then, we utilized co-expression analysis to obtain irlncRNAs that were co-expressed with IRGs (| *r* | > 0.6, *p* < 0.001). A total of 364 irlncRNAs were identified, of which 275 were distinguished as prognostic via univariate Cox analysis (*p* < 0.01).

### *Construction of *the* prognostic IRGs and irlncRNAs signature model*

**Fig. 2:**
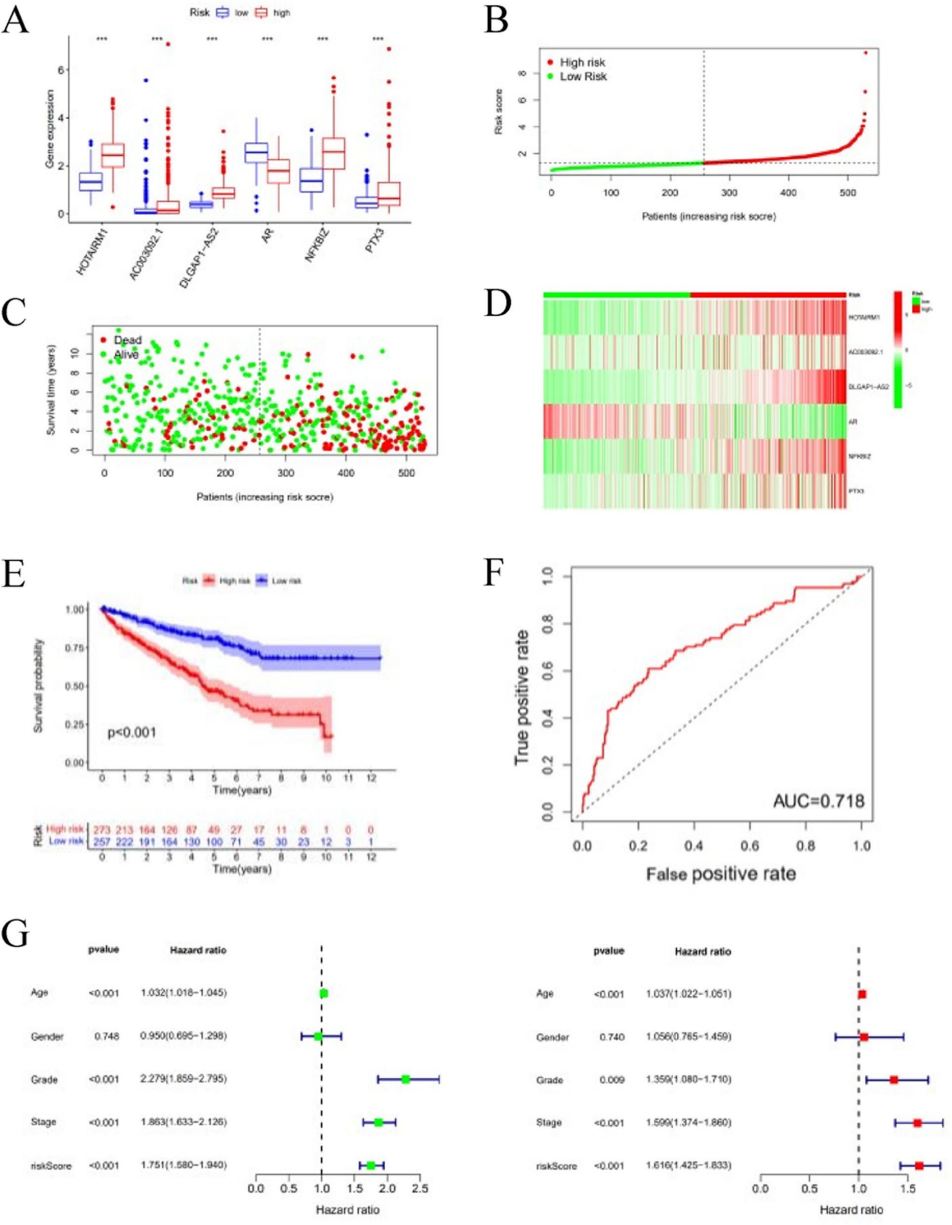
Risk model construction and its related analysis. **A** Expression levels of the genes and lncRNAs involved in model construction, ***p < 0.001. **B–C** Distribution of the risk scores, survival status, and the heat maps of three hub lncRNAs and three hub mRNAs. **E–F** Survival curve and ROC curve analysis. **G** Univariate and multivariate Cox regression analyses for patients with ccRCC

The previously acquired 200 IRGs and 275 irlncRNAs were used to construct the LASSO Cox regression model regarding the prognosis of ccRCC (Fig. 1B–C). We integrated the expression profiles of 3 hub genes (AR, TREM-1, PTX3) and 3 hub lncRNAs (HOTAIRM1, AC003092.1, DLGAP1-AS2) involved in model construction (Fig. 2A). The risk score was calculated by multiplying the expression level of each gene or lncRNA and its corresponding coefficient. The 530 ccRCC samples were divided into low- and high-risk groups based on the median risk score (Fig. 2B–D). Kaplan Meier analysis showed that the long-term survival time of patients with ccRCC in the high-risk group was shorter than that in the low (Fig. 2E). ROC curve analysis showed that the AUC for 3-year survival was 0.718 (Fig. 2F).

### *Model validation *and* clinical relationship assessment*

Both univariate and multivariate Cox regression results showed that the tumor stage, tumor grade, and the risk score of this model were all associated with the prognosis of ccRCC (Fig. 2G). These results demonstrated that our model could be used as the independent prognostic factor for ccRCC. The relationship between risk groups and clinicopathological characteristics noted that the risk score was correlated with survival status, tumor grade, and tumor stage but not with the age or gender of the patients (Table 1).

**Table 1:** Relationship between risk groups and clinicopathological characteristics in 524 ccRCC patients

Characteristics	Total	High risk	Low risk	*χ* ^2^	*p* value
Survival state					
Alive	352	149	203	36.71	0
Dead	172	122	50		
Age (years)					
≤ 65	344	174	170		0.53
> 65	180	97	83	0.39	
Gender					
Female	185	96	89	0	1
Male	339		175	164	
Grade					
G1	14	7	7	17.85	0.0013
G2	225	104	121		
G3	206	103	103		
G4	74	52	22		
GX	5	5	0		
Stage					
Stage I	263	120	143	10.92	0.012
Stage II	56	30	26		
Stage III	123	67	56		
Stage IV	82	54	28		

*ccRCC* Clear cell renal cell carcinoma

### *GO, KEGG, and PPI *analyses

**Fig. 3:**
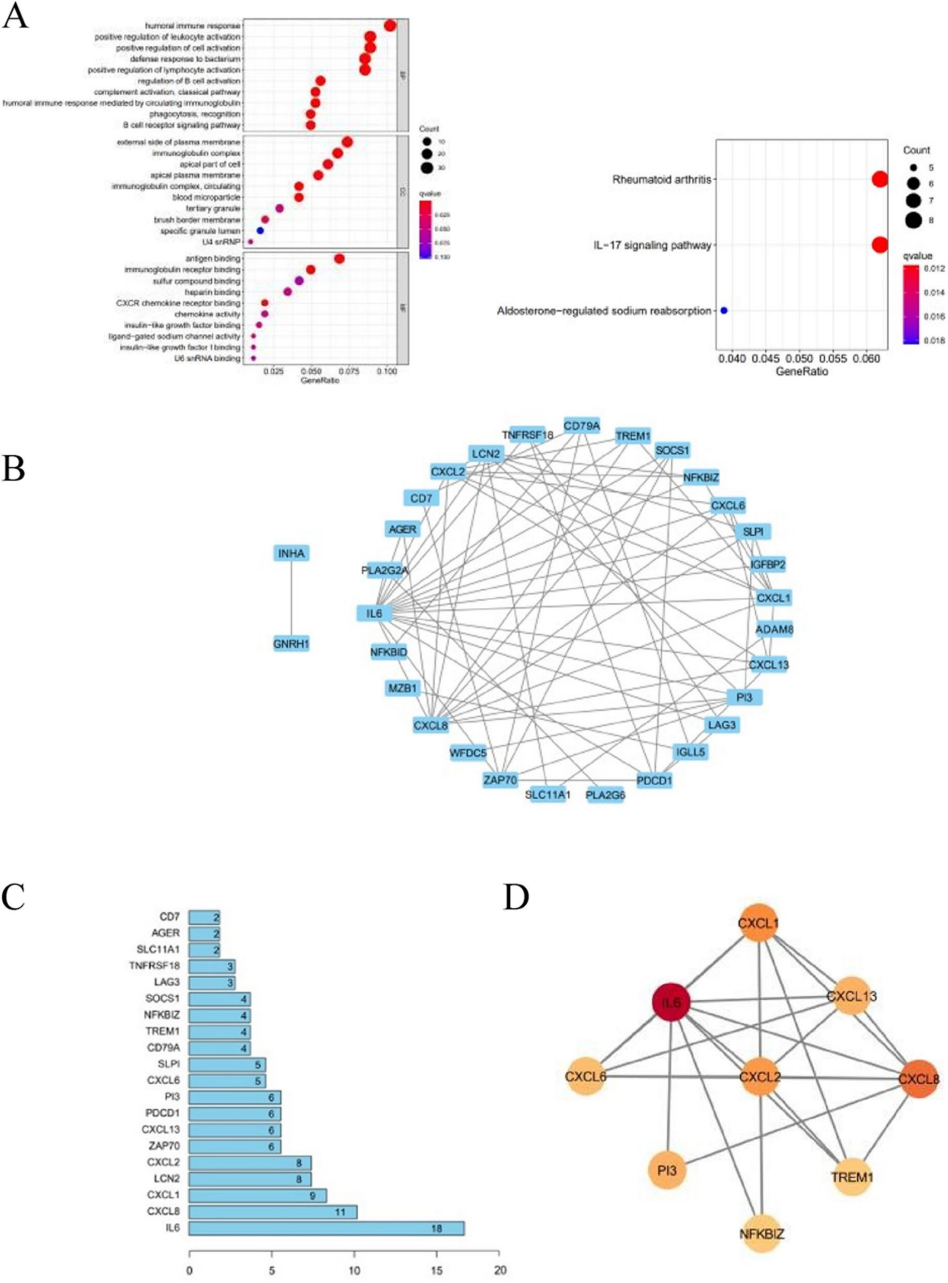
The enrichment analysis. **A** GO and KEGG enrichment analysis of 503 DEGs. **B** Construction of a PPI network that includes 36 nodes and 64 edges. **C** The number of nodes in the top 20 immune-related DEGs. **D** A significant module was selected using the MCODE plugin of Cytotype software

We conducted differential gene expression analysis and obtained 503 DEGs, including 66 IRGs. Then, we performed GO and KEGG analysis of these DEGs (Fig. 3A). To search for immune-related DEGs, we selected genes enriched via GO involved in the immune response, including B-cell activation, B-cell mediated immunity, T-cell activation, etc. Next, we constructed the PPI network for these selected immune-related DEGs using string and cytoscape software (Fig. 3B). The number of nodes in the top 20 immune-related DRGs is shown in Fig. 3C. Eventually, we selected a significant module from the MCODE plugin analysis that included the following genes: IL-6, CXCL1, CXCL13, CXCL8, CXCL2, CXCL6, PI3, NFKBIZ and TREM-1 (Fig. 3D).

### Correlation between TREM-1 expression and ccRCC

**Fig. 4:**
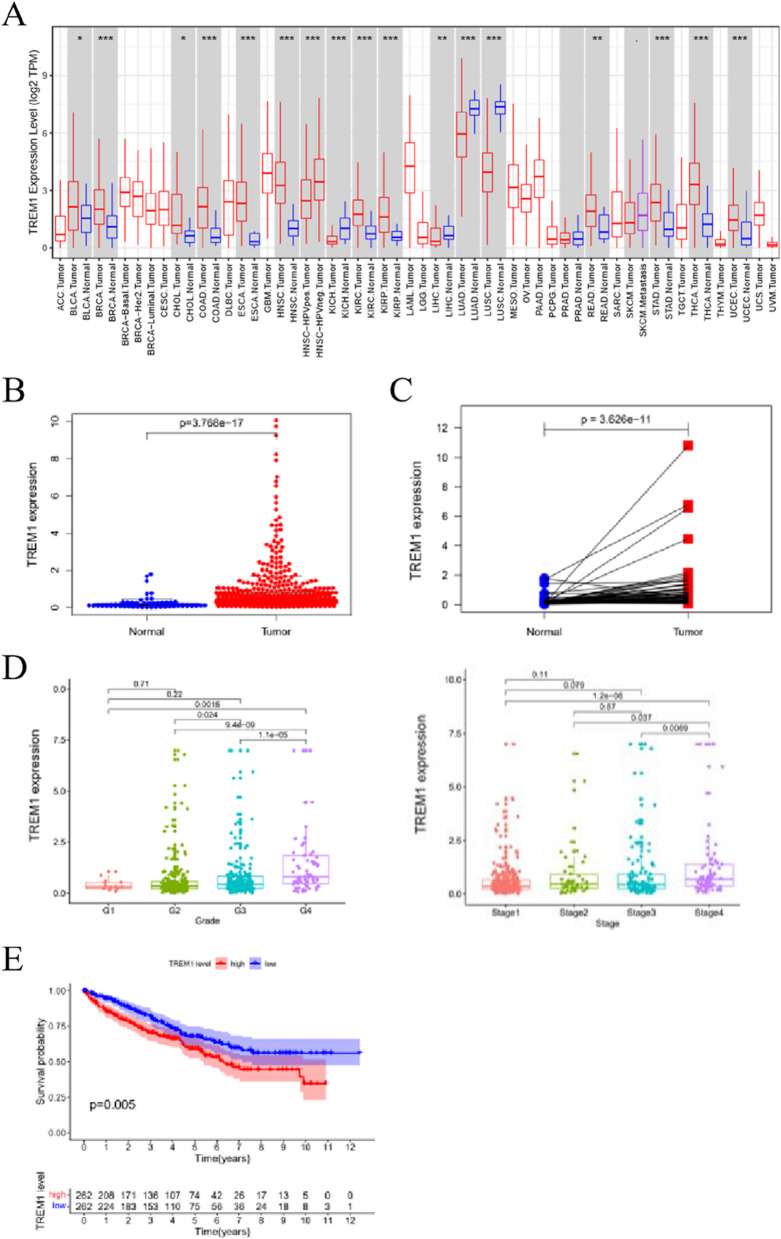
The relationship between TREM-1 and ccRCC in TCGA. **A** The TREM-1 expression profiles in different cancers, *p < 0.05, **p < 0.01, ***p < 0.001. **B–D** Expression levels of TREM-1 in patients with ccRCC. **E** Survival curve analysis

We analyzed the clinical relationship between the nine immune-related DEGs and ccRCC according to the ranking order obtained from the ENCODE results and selected the hub gene TREM-1. The oncomine database analysis revealed that TREM-1 was highly expressed in different types of cancer, including ccRCC (Fig. 4A). We further validated the relationship of TREM-1 to ccRCC using the TCGA database, and the results were similar to before (Fig. 4B–C). The TREM-1 levels were gradually upregulated along with the tumor grades and stage 3 to stage 4 in ccRCC (Fig. 4D). Furthermore, the analysis of the clinical characteristics revealed that TREM-1 was correlated with survival status, grade, and TNM stage in ccRCC (Table 2). The survival analysis showed that ccRCC patients with high TREM-1 expression have a shorter survival time than those with low expression (Fig. 4E). Univariate and multifactorial Cox regression results indicated that TREM-1 is an independent prognostic factor for ccRCC (Table 3).

**Table 2:** Relationship between TREM-1 expression level and clinicopathological characteristics in 524 KIRC patients

Characteristics	Total	High exp	Low exp	*χ* ^2^	*p* value
Survival state					
Alive	352	162	190	7.63	5.70E − 03
Dead	172	102	70		
Age(y)					
≤ 65	344	173	171	0	1
> 65	180	91	89		
Gender					
Female	185	98	87	0.62	0.43
Male	339	166	173		
Grade					
G1	14	5	9	33.01	0
G2	225	94	131		
G3	206	106	100		
G4	74	58	16		
GX	5	1	4		
Stage					
Stage I	263	113	150	21.13	1.00E − 04
Stage II	56	29	27		
Stage III	123	63	60		
Stage IV	82	59	23		

*ccRCC* Clear cell renal cell carcinoma, *High exp* High TREM1 expression, *Low exp* low trem1 expression

**Table 3:** Univariate and multivariate COX regression of TREM-1

Characteristics	Univariate analysis		*p* value	Multivariate		
	HR	95% CI		HR	95% CI	*p* value
Age	1.03	1.02–1.05	3.27E − 06	1.03	1.02–1.05	7.46E − 06
Gender	0.95	0.7–1.3	0.747514	0.94	0.68–1.29	0.700349
Grade	2.28	1.86–2.79	2.32E − 15	1.49	1.18–1.88	0.000682
Stage	1.86	1.63–2.13	2.32E − 20	1.65	1.42–1.91	9.72E − 11
TREM-1	1.07	1.03–1.1	5.96E − 05	1.04	1–1.07	0.029218

### Correlation of TREM-1 with immune infiltration and their gene markers in ccRCC

**Fig. 5:**
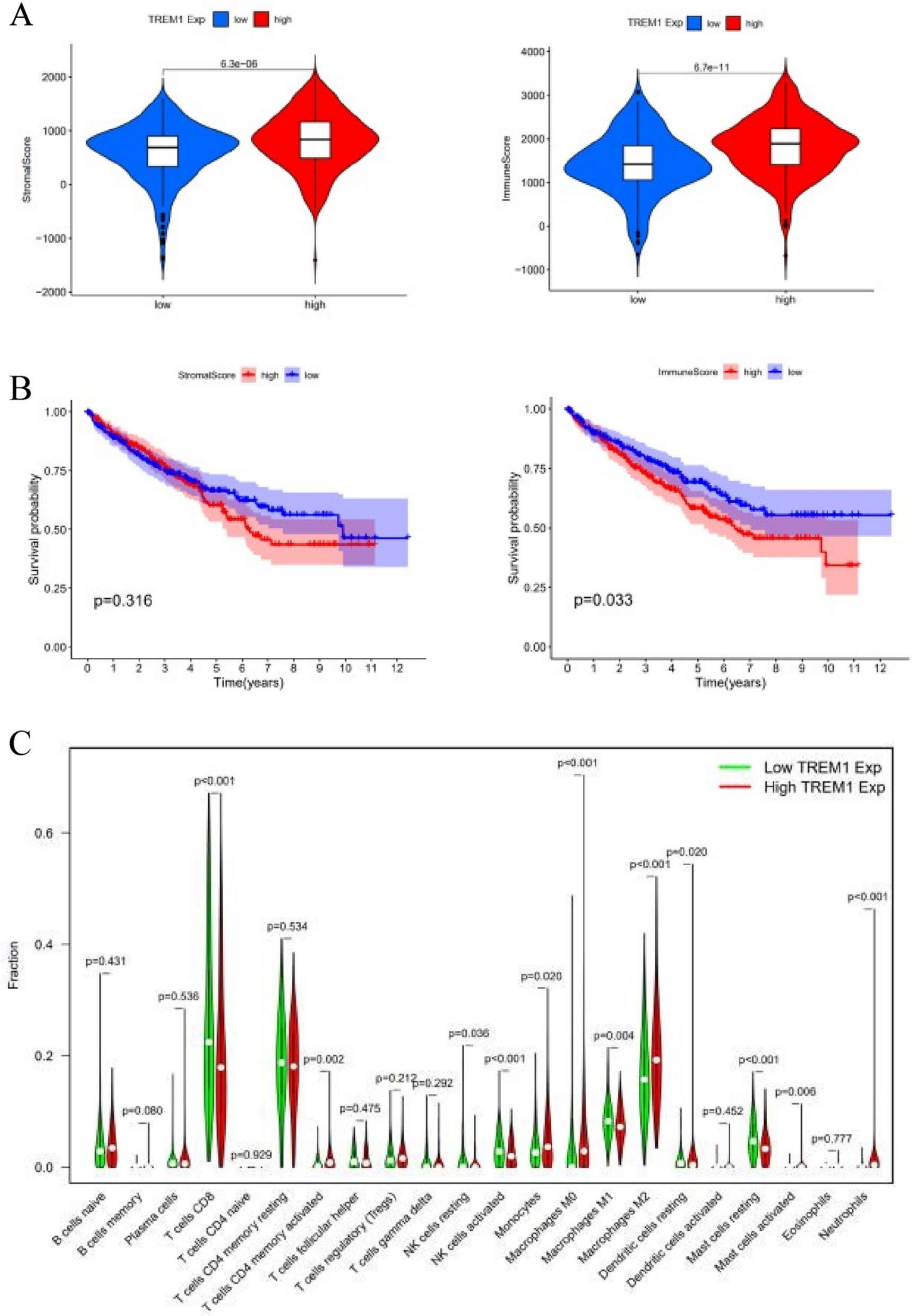
Tumor microenvironment scores and survival analysis. **A** The immune scores in TME. **B** The survival analysis of the immune score. **C** Violin plot of 22 types of immune cells

The ESTIMATE results revealed that the presence of stromal cells and immune cells in the high-TREM-1 expression group was more abundant than the group with the low expression (Fig. 5A). The survival analysis showed a negative correlation between the immune score and survival time (Fig. 5B). The CIBERSORT analysis results displayed higher contents of activated memory CD^4+^ T cells, monocytes, M0 and M2 macrophages, resting dendritic cells, activated resting mast cells, and neutrophils in the high-TREM-1 expression group than in the low-TREM-1 expression group. In contrast, a low expression of TREM-1 yielded higher concentrations of CD^8+^ T cells, resting NK cells, activated NK cells, M1 macrophages, and resting mast cells (Fig. 5C). In addition, the different abundances of the infiltrated immune cells between the high- and low-TREM-1 expression groups were further confirmed through the correlation analysis conducted between the different marker subsets of immune cells and TREM-1 [25]. These findings validate that TREM-1 is strongly linked with innate immunity since the major innate immune cell types such as dendritic cells, neutrophils, natural killer cells, and macrophages were strongly associated with TREM-1 expression (Table 4).

**Table 4:** Relationship between TREM-1 and immune cell markers

Markers	TREM-1 expression		*p* value
	Low median (IQR)	High median (IQR)	
CD8 + T cells			
CD8A	4.02 (1.78, 8.91)	4.83 (2.08, 11.86)	0.1521
CD8B	1.51 (0.67, 3.78)	1.77 (0.65, 4.72)	0.2803
T cell (general)			
CD3D	5.91 (3.10, 12.27)	7.17 (3.22, 14.14)	0.0961
CD3E	6.18 (3.15, 10.82)	6.88 (3.40, 13.22)	0.0758
CD2	8.48 (4.24, 14.31)	9.87 (4.62, 18.40)	0.1377
B cells			
CD19	0.09 (0.05, 0.18)	0.12 (0.07, 0.32)	< 0.0001
CD79A	0.81 (0.40, 1.87)	1.10 (0.51, 3.62)	0.0009
Monocytes			
CD86	3.00 (1.77, 4.76)	5.29 (3.03, 7.78)	< 0.0001
CSF1R	14.07 (8.05, 21.92)	21.14 (12.12, 31.4)	< 0.0001
TAM			
CCL2	25.13 (12.21,45.31)	33.27 (19.05,69.23)	< 0.0001
CD68	1.14 (0.60, 1.80)	1.50 (0.87, 2.48)	0.0001
IL10	0.22 (0.11, 0.40)	0.45 (0.22, 0.84)	< 0.0001
M1 macrophages			
NOS2	0.72 (0.39, 1.27)	0.65 (0.36, 1.12)	0.1412
IRF5	4.61 (3.07, 6.16)	5.23 (3.69, 7.01)	0.0004
PTGS2	0.84 (0.41, 1.76)	1.27 (0.61, 2.84)	< 0.0001
M2 macrophages			
CD163	7.12 (3.47, 13.83)	14.36 (6.17, 24.42)	< 0.0001
VSIG4	7.90 (4.31, 13.08)	16.02 (7.40, 28.31)	< 0.0001
MS4A4A	5.50 (3.14, 9.09)	9.31 (4.81, 14.98)	< 0.0001
Neutrophils			
CD66b	0.01 (0.00, 0.02)	0.01 (0.00, 0.02)	0.5579
CD11b	2.88 (1.71, 4.19)	4.3 (2.63, 6.25)	< 0.0001
CCR7	0.9 (0.48, 1.48)	1.21 (0.65, 2.11)	< 0.0001
NK cells			
KIR2DL1	0.09 (0.04, 0.17)	0.08 (0.03, 0.14)	0.066
KIR2DL3	0.06 (0.03, 0.12)	0.05 (0.02, 0.10)	0.0506
KIR2DL4	0.23 (0.10, 0.42)	0.25 (0.10, 0.51)	0.319
KIR3DL1	0.07 (0.03, 0.15)	0.06 (0.02, 0.12)	0.0159
Dendritic cell			
HLA-DQB1	46.91 (21.45, 87.60)	53.96 (26.37, 90.47)	0.1491
HLA-DPB1	118.82 (64.46, 193.7)	160.33 (90.71, 233.04)	0.0007
HLA-DRA	757.63 (411.78, 1195.93)	986.52 (554.79, 1494.01)	0.0001
BDCA-1	0.74 (0.40, 1.39)	0.94 (0.52, 1.49)	0.041
BDCA-4	37.31 (21.78, 51.76)	37.64 (23.61, 50.55)	0.8289
CD11c	2.98 (1.40, 4.91)	4.74 (2.47, 7.86)	< 0.0001
Th1			
TBX21	0.85 (0.44, 1.37)	0.7 (0.37, 1.23)	0.9998
STAT4	1.17 (0.66, 1.67)	1.24 (0.74, 1.79)	0.1184
STAT1	33.78 (22.56, 50.48)	40.61 (27.01, 62.58)	0.0003
INF-γ	0.19 (0.06, 0.49)	0.21 (0.07, 0.63)	0.3898
TNF-α	0.30 (0.14, 0.58)	0.45 (0.25, 0.79)	< 0.0001
Th2			
GATA3	1.42 (0.75, 3.86)	1.62 (0.86, 3.62)	0.3998
STAT6	35.79 (28.29, 43.21)	36.03 (28.94, 41.36)	0.6576
STAT5A	5.88 (4.40, 7.42)	6.7 (5.28, 8.34)	< 0.0001
IL13	0.01 (0.00, 0.02)	0.01 (0.00, 0.03)	0.0709
Tfh			
BCL6	10.75 (7.77, 14.61)	12.12 (8.86, 16.17)	0.0027
Th17			
STAT3	26.71 (21.49, 31.65)	29.45 (24.78, 34.8)	< 0.0001
Tregs			
FOXP3	0.49 (0.26, 1.04)	0.77 (0.33, 1.69)	< 0.0001
CCR8	0.09 (0.02, 0.22)	0.14 (0.05, 0.35)	0.0007
STAT5B	17.22 (14.61, 20.33)	16.4 (13.61, 19.01)	0.0053
TGF-β	37.90 (24.11, 49.6)	38.57 (25.95, 52.5)	0.2186
T cell exhaustion			
PD-1	0.68 (0.35, 1.96)	1.17 (0.42, 2.59)	0.0051
CTLA4	0.33 (0.13, 0.83)	0.56 (0.23, 1.30)	0.0002
LAG3	1.02 (0.49, 2.72)	1.32 (0.60, 3.48)	0.071
TIM-3	13.66 (5.61, 27.42)	14.81 (7.57, 27.71)	0.1604
GZMB	3.02 (1.45, 5.06)	2.77 (1.47, 5.25)	0.8507

### *TREM-1 showed *correlation* IHC*

**Fig. 6:**
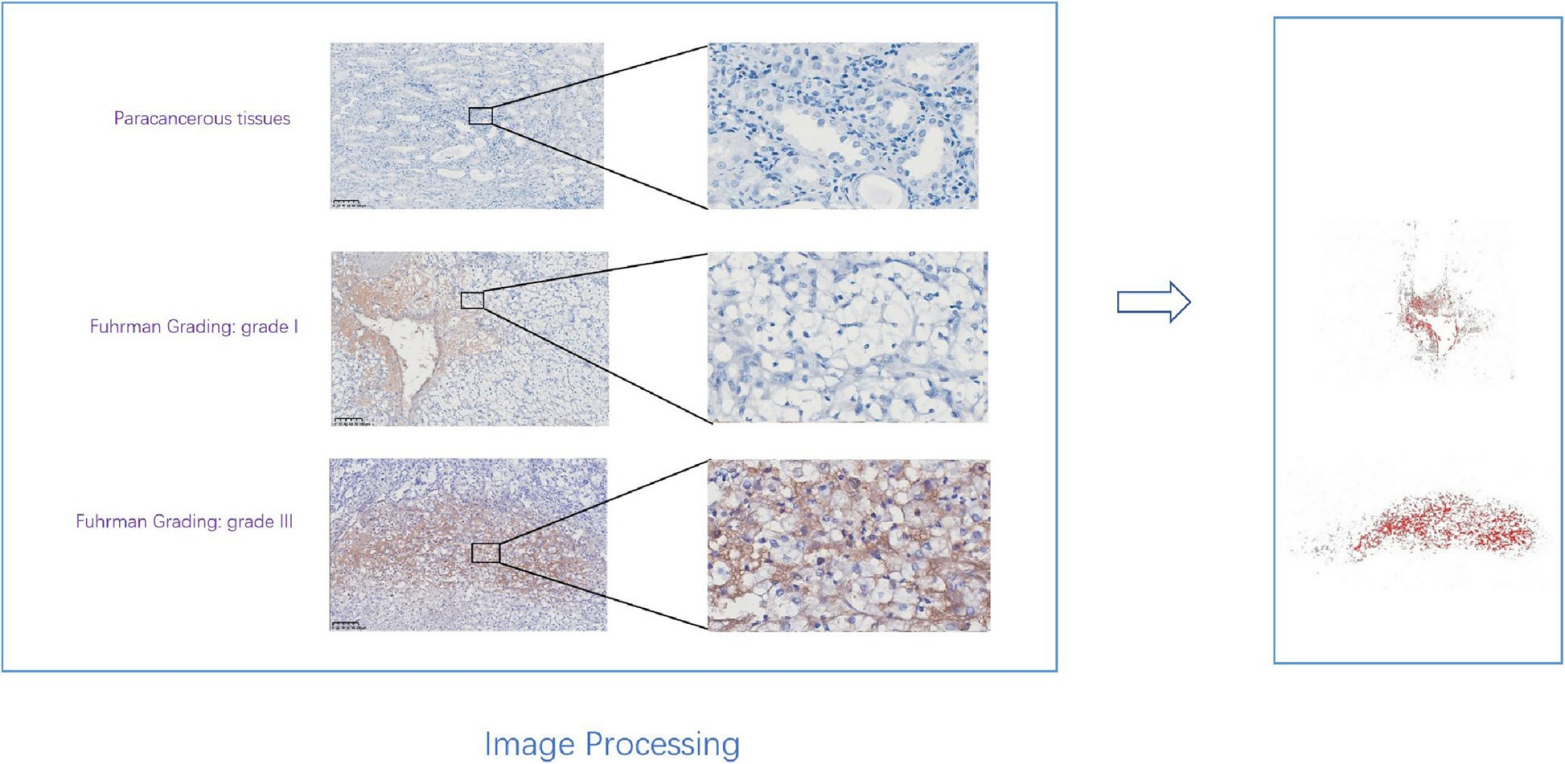
Immunohistochemical analysis of TREM-1 expression in renal clear cell carcinoma

We found that uniform cytoplasmic and membrane staining showed positive expression of TREM-1. In benign tissues, TREM-1 expression was very low, but high in tumor tissue areas. And TREM-1 expression increased with increasing Fuhrman grade. OD values: Grade 3 vs Grade 1: 0.308 vs 0.199 (Fig. 6).

### *Prediction of *the* function of TREM-1*

**Fig. 7:**
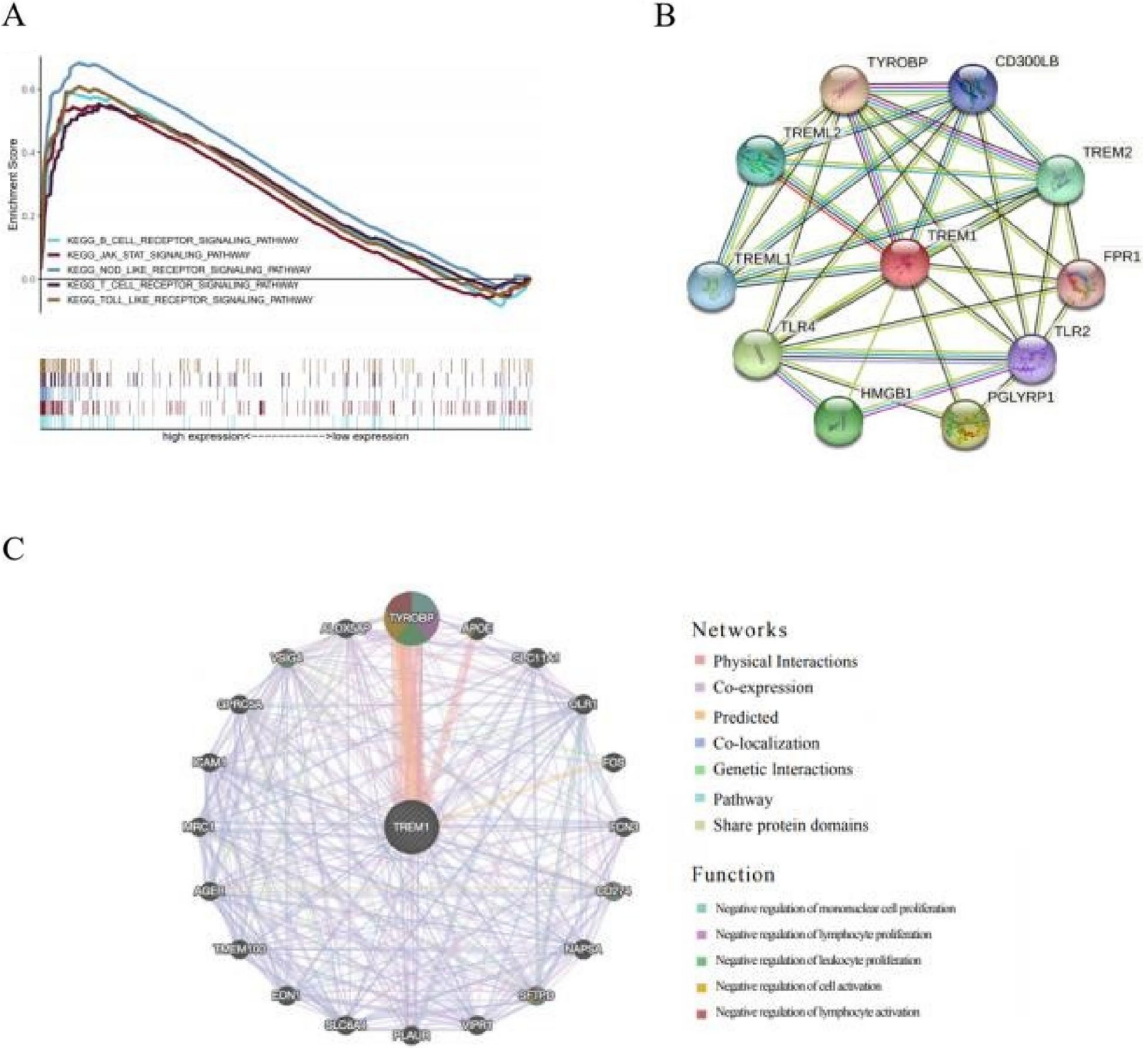
The GSEA and PPI enrichment. **A** The GSEA results. **B–C** The functional interactions related to TREM-1 in string and GeneMANIA

**Fig. 8:**
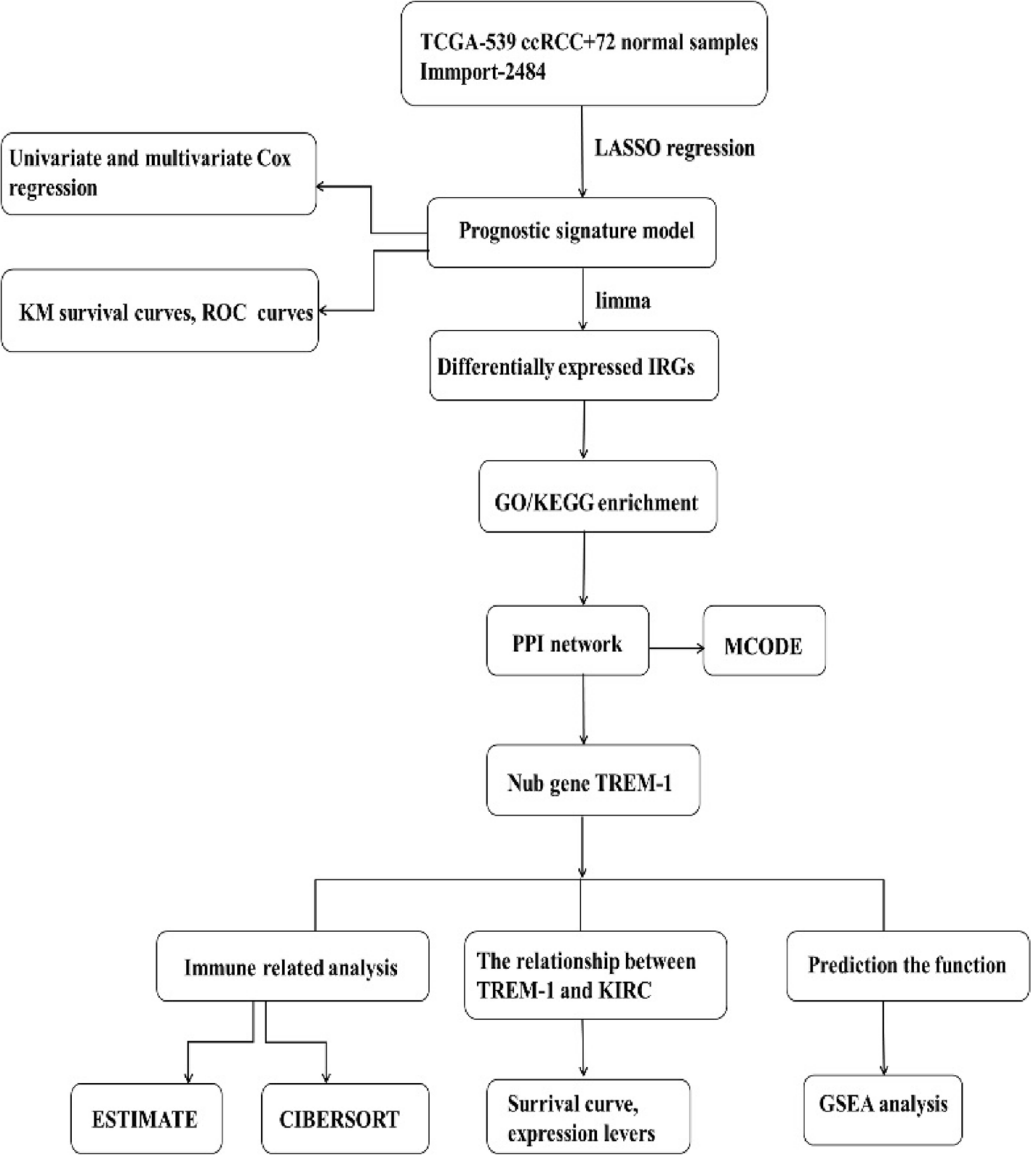
Flow chart of bioinformatics analysis in this paper

GSEA analysis showed that TREM-1 was involved in various immune-related pathways, including B-cell receptor, JAK-STAT, NOD-like receptor, Toll-like receptor, and T-cell receptor signaling pathway (Fig. 7A). Moreover, the string and GeneMANIA results showed that the function of TREM-1 and its associated molecules (TYROBP, CD274, SFTPD, and VSIG4) were primarily related to the negative regulation of mononuclear cell, lymphocyte and leukocyte proliferation, and the negative regulation of lymphocyte activation (Fig. 7B-C). These results indicate that TREM-1 is closely related to immune infiltration. Flow chart of bioinformatics analysis in this paper (Fig. 8).

## Discussion

The TME could affect the prognosis of patients in the primary location, as there is complicated dynamic crosstalk among neoplastic and non-neoplastic cells, including adjacent stromal cells and infiltrating immune cells. In addition, immune and stromal scores were significantly associated with the expression of TREM-1. Our findings showed that only the group with a higher immune score had a poor survival prognosis, consistent with those of Wu et al. [26]. Interestingly, unlike previous studies of gastric cancer [27], clear cell renal cell carcinoma [28], and glioblastoma [29], higher stromal and immune scores indicate better OS in patients, suggesting that TME scores may have different prognostic values in different tumor types. Subsequently, we identified that one of the immune-related hub gene responsible for this difference is TREM1 and is gradually upregulated with increasing pathologic stages and grades. Therefore, TREM1 might be identified as a novel and potential biomarker that could be of interest for further research in ccRCC.

Previous studies have shown that macrophages, which play an essential role in modulating the tumor immune microenvironment, are generally divided into two categories: M1 macrophages of the classical activation pathway and M2 macrophages of the alternative activation pathway. In this study, we found that compared to M1 macrophages, the content of M2 macrophages was more abundant in the high TREM-1 expression group. However, the functions of the two are different, with M1 macrophages inhibiting tumor progression and M2 macrophages having a tumor-promoting phenotype [30, 31]. Furthermore, Yoshihiro et al. revealed that TAMs, especially M2-polarized TAMs, contribute to the activation of STAT3, which can promote cancer cell survival, angiogenesis, and immunosuppression in the tumor microenvironment [32]. The content of Tregs is also increased in high TREM-1 group, which could produce immunosuppressive cytokines and immune-inhibitory receptors to disturb the activation of anti-tumor T cell responses, leading to a worse prognosis.

We further found that TREM-1 levels in ccRCC correlate with T cell exhaustion markers (PD-1 and CTLA-4). CTLA-4 and PD-1, which have been widely studied in malignant tumors, can be converted into immune regulation of TME [33]. PD-1 signaling inhibits Akt phosphorylation by preventing CD28-mediated activation of phosphatidylinositol 3-kinase (PI3K). The differential regulation of PI3K activation by the ligation of PD-1 and CTLA-4 led to different cell phenotypes, while TREM-1’s conduction pathway can lead to downstream signal transduction of PI3K [34]. Hence, TREM-1 might play a critical role in regulating the tumor microenvironment in ccRCC and may influence the prognosis of patients.

To explore the underlying molecular mechanism of TREM-1, we further conducted GSEA and constructed the PPI network. GSEA results revealed that the pathways involving TREM-1 were mainly enriched in the B-cell receptor, JAK/STAT, NOD-like receptor, T-cell receptor, and Toll-like receptor signaling pathways. These pathways have previously been reported to be involved in the immune response in ccRCC [35–37]. As reported, the effect of inhibiting the JAK/STAT signaling pathway on the suppression of immune activity appears promising and could become a strategy to prevent tumor progression [38]. The top 10 related genes that may have a similar function in ccRCC were predicted by constructing a string network. A recent study uncovered TREM2/APOE/C1Q-positive macrophage infiltration as a potential prognostic biomarker and a candidate therapeutic target [39]. Further research revealed that TYROBP, HMGB1, and TLR4 were also significantly associated with the progression of ccRCC (40).

However, this research had a few limitations. First, although we confirmed that TREM-1 was highly expressed in tumor tissue compared with benign tissue, and the expression was positively correlated with Fuhrman grade, by immunohistochemistry experiment. However, due to the limitation of the small number of tumor tissue sections, the expression and distribution of TREM-1 and the invasion of immune cells were not simultaneously investigated. Second, the bioinformatics analysis needs to be supported by future biological experiments that involve in vivo and in vitro methods. In summary, these results indicate that TREM-1 may have the potential to drive the progression of ccRCC. Therefore, this gene may serve as an independent prognostic predictor and be a candidate novel biomarker for ccRCC diagnosis.

## Data Availability

All data generated or analyzed during this study are included in this published article.
